# Enhanced Production of Recombinant Secretory Proteins in *Pichia* *pastoris* by Optimizing Kex2 P1’ site

**DOI:** 10.1371/journal.pone.0075347

**Published:** 2013-09-19

**Authors:** Song Yang, Ye Kuang, Hongbo Li, Yuehong Liu, Xiaoyan Hui, Peng Li, Zhiwu Jiang, Yulai Zhou, Yu Wang, Aimin Xu, Shiwu Li, Pentao Liu, Donghai Wu

**Affiliations:** 1 The Key Laboratory of Regenerative Biology and The Guangdong Provincial Key Laboratory of Stem Cell and Regenerative Medicine, Guangzhou Institute of Biomedicine and Health, Chinese Academy of Sciences, Guangzhou, China; 2 Department of Biomedical Engineering, School of Pharmaceutical Sciences, Jilin University, Changchun, China; 3 The Key Laboratory of Research and Utilization of Ethnomedicinal Plant Resources of Hunan Province, Department of Life Sciences, Huaihua College, Huaihua, China; 4 Department of Medicine, The University of Hong Kong, Hong Kong, China; 5 Department of Pathology, University of Florida, Gainesville, Florida, United States of America; 6 The Wellcome Trust Sanger Institute, Cambridge, United Kingdom; Central China Normal University, China

## Abstract

*Pichia* *pastoris* is one of the most widely used expression systems for the production of recombinant secretory proteins. Its universal application is, however, somewhat hampered by its unpredictable yields for different heterologous proteins, which is now believed to be caused in part by their varied efficiencies to traffic through the host secretion machinery. The yeast endoprotease Kex2 removes the signal peptides from pre-proteins and releases the mature form of secreted proteins, thus, plays a pivotal role in the yeast secretory pathways. In this study, we found that the yields of many recombinant proteins were greatly influenced by Kex2 P1' site residues and the optimized P1’s amino acid residue could largely determine the final amount of secretory proteins synthesized and secreted. A further improvement of secretory yield was achieved by genomic integration of additional Kex2 copies, which again highlighted the importance of Kex2 cleavage to the production of recombinant secretory proteins in *Pichia* yeast.

## Introduction

Protein based biopharmaceuticals make up the largest and fastest growing part of global top selling drugs [1,2]. *Pichia* *pastoris* is one of the most commonly used expression hosts for production of heterologous secretory proteins [3], thanks mainly to a highly efficient and tightly regulated expression system based on the promoter of the alcohol oxidase 1 gene (*AOX1*), high levels of protein products being secreted into almost protein-free media as well as its capacity of carrying out correct folding and post-translational modification for mammalian proteins [4-8]. Genetic engineering on this strain to optimize the yield of expression, including analysis of *Pichia* *pastoris* genome [9-11], transcriptome [12-15], and proteome [16], as well as glycoengineering [17-19], promoters and regulatory factors engineering [20-24], has always been the hot and practical topic in the area. Although extensive efforts have been made, the secretory protein yields are still highly variable due to the inherent properties of the foreign proteins of interest [8,25]. Although there are many reports of secretion of recombinant proteins with yields up to the range of grams per liter [26-28], for apparently obvious reasons, cases of low secretory yields or complete failure are seldom published. Recently it is reported that the trafficking of folded proteins through secretion machinery, rather than transcription and translation, is most likely the rate-limiting step in the final yield of recombinant proteins [25]. However, the strategies to improve the efficiency of this secretion machinery and the specific components within this complex system that may serve as a viable target for engineering remain elusive.

**Figure 1:**
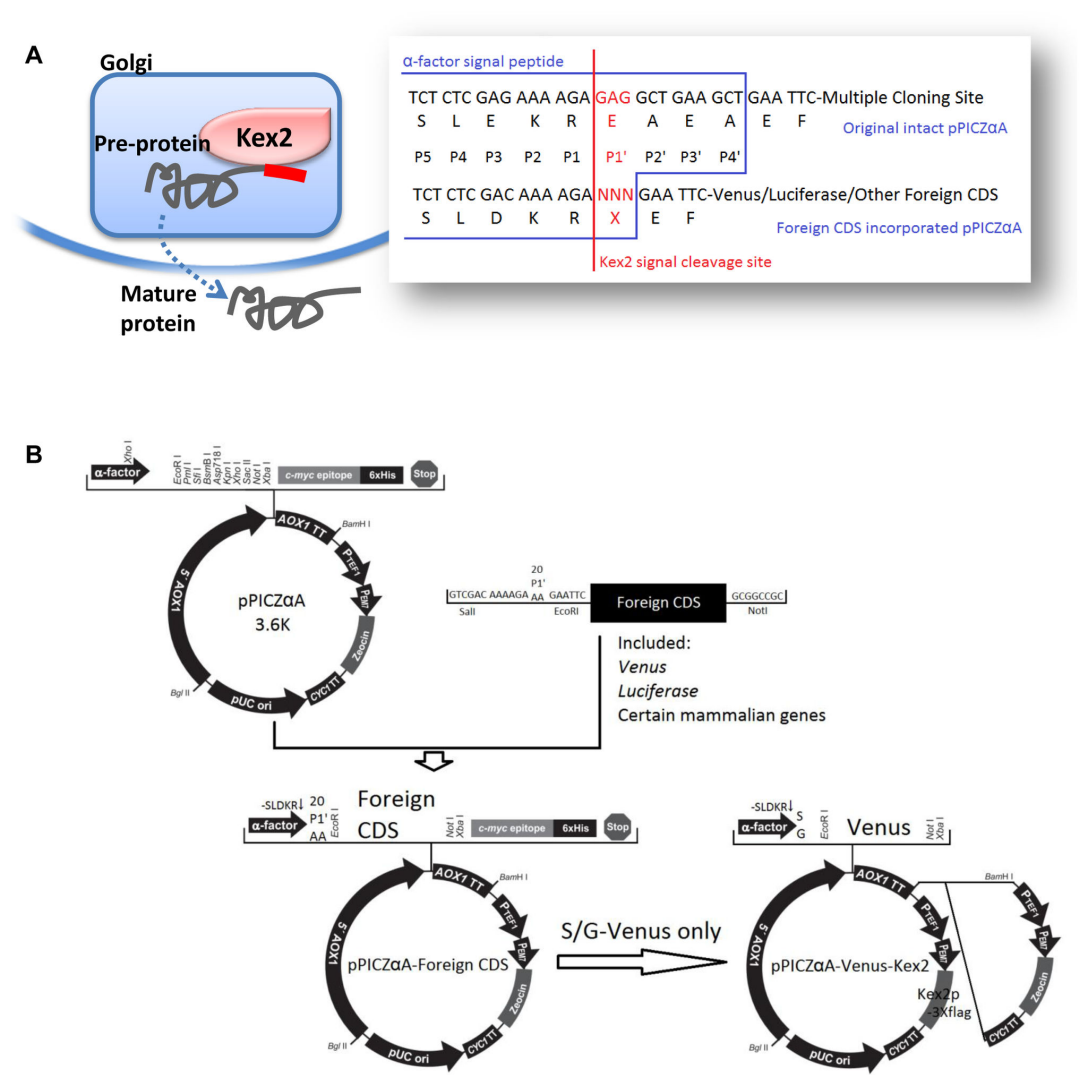
The scheme of rationale and the construction of a library of vectors: (A) the physiological role and cleavage site of Kex2; (B) the construction of pPICZαA-CDS libraries with all 20 natural amino acids at Kex2 P1’ site and the pPICZαA-S/G-Venus-Kex2 with additional *Kex2* copy.

The yeast *KEX2* gene encodes a Ca^2+^ dependent serine endoprotease [29,30] which cleaves the yeast endogenous pre-proteins for maturation in a site-specific manner [31,32] (paired dibasic sites in target peptides). The cleavage process removes the signal peptides of pre-proteins in the late Golgi (Figure 1A), which facilitates the subsequent entry of the mature proteins toward the secretion vesicles and thus represents a key step in the yeast secretion pathways [31,33,34]. Moreover, *in vivo* positive correlation between Kex2 cleavage and yeast secretory rate has been reported [35], indicating that optimization of Kex2 cleavage might represent an efficient way to improve yeast secretion productivity. As an endoprotease with a relatively fixed cleavage site, the site specificity of Kex2 has been under intense investigation [34]. The most stringent and crucial selectivity occurs at P1 site, where only Arginine is accepted [34,36], while at P2 site, basic residues such as Lysine or Arginine are recognized equally well [35] (Figure 1A). At P4 site, dual recognition of both aliphatic and basic side chains are acceptable [37] (Figure 1A). In contrast, on the other side of the Kex2 scissile bond, the substrate residue specificity is relatively less selective, except that bulky side chains are disfavored at P1’ site [38] (Figure 1A) according to previous reports based on *in vitro* enzymatic characterizations with short synthetic peptide as substrates [39,40], which may or may not truly reflect the situations *in vivo*.

To determine whether Kex2 cleavage efficiency influences the secretion levels of the heterologous proteins, we have developed a set of recombinant library yeast vector system with all twenty naturally occurring amino acid present at the Kex2 P1’ site. Reporter genes (*Venus* and *luciferase*) and several mammalian proteins were tested in this library system (Figure 1B). We demonstrated that optimization at the Kex2 P1’ site residue substantially enhanced the production of the foreign secretory proteins. In addition, additional *Kex2* copies introduced into yeast genome further increased the secretion yield, which again demonstrated the feasibility of augmenting secretory productivity via enhancement of the Kex2 cleavage.

## Materials and Methods

### Strains, plasmids, and reagents

*Escherichia coli* TOP10 strain, *Pichia* *pastoris* X-33 strain, pPICZαA secretory expression vector, yeast nitrogen base (YNB), D-sorbitol, D-biotin and BCA protein concentration assay kit were purchased from Invitrogen (CA, USA). Tryptone and yeast extract were purchased from Oxoid (Hampshire, England). Polyethylene glycol (PEG) 3350 and lithium chloride (LiCl) were purchased from Sigma-Aldrich (MO, USA). Sonicated single stranded salmon sperm DNA was purchased from Genmed (MA, USA). Zeocin was purchased from Invivogen (CA, USA). Steady-Glo® Luciferase Assay System was purchased from Promega (WI, USA). Plasmid miniprep kit, DNA recovery/purification kit, pMD20-T cloning vectors, restriction endonucleases, DNA polymerases for PCR reaction and T4 DNA ligase were purchased from Takara (Guangzhou, China). Primers synthesis and DNA sequencing service were provided by Invitrogen (Shanghai, China). *Escherichia coli* TOP10 single colonies were selected on normal (for pMD20-T cloning) or low salt (for pPICZαA cloning) LB agar plates (1% tryptone, 0.5% yeast extract, 1% (normal) or 0.5% (low salt) NaCl and 1.5% agar) with corresponding antibiotics. Newly generated *Pichia* *pastoris* transformants were initially selected on YPD plates (2% peptone, 1% yeast extract, 2% dextrose, 2% agar) with 100 µg/ml zeocin, then on YPD plates with increasing doses of zeocin (from 200, 500 to 1000 µg/ml) to determine the copy number of integrants. For methanol induced expression, the *P* *. pastoris* recombinants were first grown in BMGY (1% yeast extract, 2% peptone, 100 mM potassium phosphate (pH 6.0), 1.34% YNB, 4×10^-5^% biotin, 1% glycerol) medium to reach higher biomass and then induced in BMMY medium which contains the same ingredients as BMGY except the replacement of glycerol with methanol, as detailed in EasySelect^TM^ *Pichia* expression kit user manual.

### Vectors construction

*Venus* coding DNA sequence (CDS), followed by kanamycin resistance gene, was amplified with primers (forward primers which covered the P1’ site were listed on Table 1 with the reverse primer of 5’-GGCTAGCGGCCGCAGACATGATAAGATACATTGATGAG-3’ which complements the 3’ terminus of kanamycin resistance gene) to place all 20 amino acids (codons were chosen according to the yeast preference [41]) at P1’ site to form a vector library. The 20 PCR amplification products were sub-cloned into pMD20-T plasmid and sequenced subsequently to ensure the sequences were correct. Then these plasmids were digested with SalI and NotI, the PCR product fragments were recovered from the agarose gel and inserted between the XhoI and NotI sites of pPICZαA, resulting in the Venus P1’ vector library (Figure 1B).

**Table 1:** Sequences of the forward primers to construct the library of vectors with 20 natural amino acids at Kex2 P1’ site, the P1’ site codons are highlighted in bold.

P1’ amino acids	Primer sequence
A	GGTCGACAAAAGA **GCT** GAATTCATGGTGAGCAAGGGCGAGGAG
C	GGTCGACAAAAGA **TGT** GAATTCATGGTGAGCAAGGGCGAGGAG
D	GGTCGACAAAAGA **GAT** GAATTCATGGTGAGCAAGGGCGAGGAG
E	GGTCGACAAAAGA **GAA** GAATTCATGGTGAGCAAGGGCGAGGAG
F	GGTCGACAAAAGA **TTT** GAATTCATGGTGAGCAAGGGCGAGGAG
G	GGTCGACAAAAGA **GGT** GAATTCATGGTGAGCAAGGGCGAGGAG
H	GGTCGACAAAAGA **CAT** GAATTCATGGTGAGCAAGGGCGAGGAG
I	GGTCGACAAAAGA **ATT** GAATTCATGGTGAGCAAGGGCGAGGAG
K	GGTCGACAAAAGA **AAA** GAATTCATGGTGAGCAAGGGCGAGGAG
L	GGTCGACAAAAGA **CTT** GAATTCATGGTGAGCAAGGGCGAGGAG
M	GGTCGACAAAAGA **ATG** GAATTCATGGTGAGCAAGGGCGAGGAG
N	GGTCGACAAAAGA **AAC** GAATTCATGGTGAGCAAGGGCGAGGAG
P	GGTCGACAAAAGA **CCA** GAATTCATGGTGAGCAAGGGCGAGGAG
Q	GGTCGACAAAAGA **CAA** GAATTCATGGTGAGCAAGGGCGAGGAG
R	GGTCGACAAAAGA **AGA** GAATTCATGGTGAGCAAGGGCGAGGAG
S	GGTCGACAAAAGA **TCT** GAATTCATGGTGAGCAAGGGCGAGGAG
T	GGTCGACAAAAGA **ACT** GAATTCATGGTGAGCAAGGGCGAGGAG
V	GGTCGACAAAAGA **GTT** GAATTCATGGTGAGCAAGGGCGAGGAG
W	GGTCGACAAAAGA **TGG** GAATTCATGGTGAGCAAGGGCGAGGAG
Y	GGTCGACAAAAGA **TAT** GAATTCATGGTGAGCAAGGGCGAGGAG

For construction of luciferase version of the library (Figure 1B), the counterpart vectors were digested with EcoRI and NotI, the plasmid backbone fragments were recovered. Luciferase CDS was amplified with 5’-GGAATTCCTCGAGATGGAAGACGCCAAAAACATAA-3’ as the forward primer and 5’-GGCGGCCGCTAGCACGGCGATCTTTCCGCCCTTC-3’ as the reverse primer and sub-cloned into pMD20-T plasmid and sequenced. Then the inserted plasmid was digested with EcoRI and NotI and the inserted fragments were recovered and ligated with vector backbone fragments originated from the Venus vector library.

For construction of other recombinant mammalian proteins version of the libraries (Figure 1B), the counterpart vectors were constructed just the same as the luciferase library, with respective gene-specific primers.

For plasmids to introduce additional Kex2 copies into yeast genome (Figure 1B), we took advantages of the zeocin resistance gene cassette from pPICZαA and chose S-Venus and G-Venus to demonstrate the utility of such approach. First, the zeocin resistance gene cassette on the pPICZαA backbone was replaced with a pair of SfiI sites through mutation PCR with 5’-GGCCATTACGGCCAAGCTTGGCCAGGGCGGCCCACGTCCGACGGCGGCCCACGG-3’ as the forward primer and 5’-GGCCGCCCTGGCCAAGCTTGGCCGTAATGGCCGGTTTAGTTCCTCACCTTGTCG-3’ as the reverse primer. Second, the *P* *. pastoris* *Kex2* CDS was cloned from yeast genome with 5’-CGGATCCACCATGTATTTGCCAGCACTTCGCTTAGC-3’ as the forward primer and 5’-GCTCGAGCAATGCCGCACGTTTGGGATGTTCATTAG-3’ as the reverse primer, sequenced and inserted into a plasmid to fuse the flag tag to C-terminus of the CDS and SfiI sites at both ends of this cassette. After elimination the endogenous SacI site with synonymous point mutation, *Kex2* CDS was then subcloned into the Zeo(R) deleted S-Venus and G-Venus between SfiI sites. Finally, Zeo(R) was reintroduced into these plasmids at BamHI site to the 3’ end of AOX1 transcription terminator.

### *Pichia* *pastoris* transformation and selection of transformants

The yeast expression library vectors were linearized by SacI digestion and transformed into *P* *. pastoris* X-33 with the lithium chloride transformation method described in EasySelect^TM^ *Pichia* expression kit user manual (Invitrogen). Transformants were initially grown on YPD plates supplemented with 100 µg/ml zeocin. After integration of the plasmid into the yeast genome was confirmed by colony PCR, resulting colonies were transferred to YPD plates with 200, 500 and 1000 µg/ml zeocin for determination of the copy number of integrants. The subsequent comparisons of secreted proteins were only made between transformants with approximately the same copy numbers as determined by the same concentration range of drug resistance against zeocin.

### *Pichia* *pastoris* cultivation and methanol induced expression

The experimental protocol from EasySelect^TM^ *Pichia* expression kit user manual to express recombinant *Pichia pastoris*h has been followed. Briefly, the selected colonies were initially cultivated (shaking vigorously at 250 rpm) in BMGY medium at 28-30°C until the value of OD_600_ reached 2 approximately. After centrifugation and removal of BMGY, cell pellets were re-suspended in BMMY to an OD_600_ of 1 to induce expression. The volume of the culture should be no more than 10-30% of the total well/tube/flask volume to ensure sufficient aeration. Methanol was added to a final concentration of 1% every 24 hours to maintain induction. Yeast culture media were sampled and assayed every 24 hours. Small-scale cultivation and expression using 96-deep-well plates (Bel-Art Scienceware, NJ, USA) were carried out as previously described [42,43] whenever high-throughput screening of the secretory productivity was needed.

### Fluorescence and luminescence assays

Before these assays, 100 µl yeast culture medium from each sample was measured for OD_600_ and the values were used to normalize fluorescence or luminescence data. For Venus fluorescence assay, the cells were sprung down and 80 µl supernatant was added to each well of 96-well solid bottom black plates (CulturPlate^TM^-96, PerkinElmer, MA, USA) and the signal measured under an excitation spectrum of 515 nm and an emission spectrum of 528 nm. For luminescence assay, 50 µl supernatant was added with equal volume of Steady-Glo® reagent in each well of 96-well solid bottom white plates (CulturPlate^TM^-96, PerkinElmer, MA, USA) and the signal measured on a Veritas ^TM^microplateluminometer from Turner Biosystems (CA, USA).

### Western blotting analysis

The yeast cell lysis and cellular total protein extraction for SDS-PAGE and western blotting were carried out as described previously [44]. The yeast culture supernatants were also collected, TCA precipitated and sampled when needed. The samples were subjected to SDS-PAGE after protein concentration determination with the BCA Protein Quantitative Analysis Kit (Shenergy Biocolor, Shanghai, China). The resulting SDS-PAGE gels were subsequently subjected to either Coomassie staining (R-250) or western blotting. For western blotting, after PVDF (Millipore) electro-transfer, the membranes were blocked, incubated with antibodies, washed and developed on Fuji medical X-ray film (Fujifilm, Tokyo, Japan) for photographing and analysis. The gray-scale intensity values were calculated by ImageJ.

### Large-scale fermentation and purification

Scale-up expression was carried out in a 2 L baffled flask. *P* *. pastoris* strain harboring the most productive P1’-stem cell factor (V-SCF, Table 2) was cultivated in 500 ml BMGY at 28-30°C with constant vigorous shaking till the value of OD_600_ reached 8.0-12.0. Cells were pelleted and re-suspended in 250 ml BMMY, then cultured for 96 hours. Methanol was added in the medium to a final concentration of 1% every 24 hours to maintain induction. Yeast culture was centrifuged; the supernatant was collected, sampled and assayed every 24 hours. The protein concentration of the supernatant was determined by Bradford assays; the supernatant collected was dialyzed against 2 L extract buffer (50 mM Tris-HCl, 200 mM NaCl, 20 mM imidazole, pH 8.0) at 4°C overnight, filtrated with 0.22 µm filter, loaded onto a 5 ml HisTrap^TM^ HP column (GE Healthcare, Piscataway, NJ) and washed with extract buffer. The protein was eluted with a gradient of 20-500 mM imidazole. The purified protein was sent for MS analysis for identification.

**Table 2:** The *P* *. pastoris* secretory expression level of foreign mammalian proteins capped with the P1’ library of 20 natural amino acids, the values of the least secreted activities of all P1’ recombinants were set as 1, and the values of the highest secreted activities of all P1’ recombinants were highlighted in bold.

	SCF	FGF16	FGF20
pPICZαA	0.01	0.001	0.002
A	54.9	2.91	1.56
C	51.12	2.25	1.44
D	29.67	3.56	1.53
E	25.49	2.96	1
F	12.53	**8.82**	1.67
G	22.61	2.79	1.47
H	20.41	3.31	1.13
I	12.78	2.58	1.73
K	25.04	3.4	1.41
L	28.43	2.48	1.24
M	65.91	1.8	1.66
N	52.7	2.37	1.45
P	26.17	8.48	1.91
Q	14.41	2.74	1.28
R	15.71	2.18	1.68
S	1	1	1
T	24.34	2.33	1.16
V	**78.56**	4.12	4.13
W	22.45	2.1	**4.54**
Y	21.23	2.55	1.57

pPICZαA: empty vector control.

### Statistical analysis

Each fluorescent/luminescent value with an error bar was presented as mean±SEM representing the average of 5-6 individual colonies for the same vector with the same antibiotic resistance range for zeocin. *P* values were calculated with *t*-test (Student’s *t*-test); the actual *P* values of all statistical significances were given on the corresponding figure legends.

## Results

### Vector Construction and Yeast Transformation

The 20 yeast recombinant Venus/luciferase expression vectors were generated by PCR amplification and TA-cloning of Venus-Kana(R) fragments with all 20 AAs at P1’ site into the original yeast secretory expression vector pPICZαA (Figure 1B). Both the Venus coding sequence (CDS) and P1’ site were confirmed by DNA sequencing (data not shown). The engineered pPICZαA vectors were transformed into the *Pichia* *pastoris* strain X-33. The resultant yeast colonies grown on YPD plates containing antibiotic zeocin (100 µg/ml) were subjected to colony PCR to confirm transformation (data not shown), and transferred to YPD plates containing 200, 500 and 1,000 µg/ml zeocin to empirically estimate the copy number of the integrants. On average, 20-30 colonies derived from each vector were tested for their integrated copy number, from which 5-6 colonies for the given vector, with zeocin resistance ranging 200-500 and 500-1,000 µg/ml, respectively, were selected for subsequent analysis of their levels of secretion.

### Cultivation and Fluorescence/luminescence Assays

**Figure 2:**
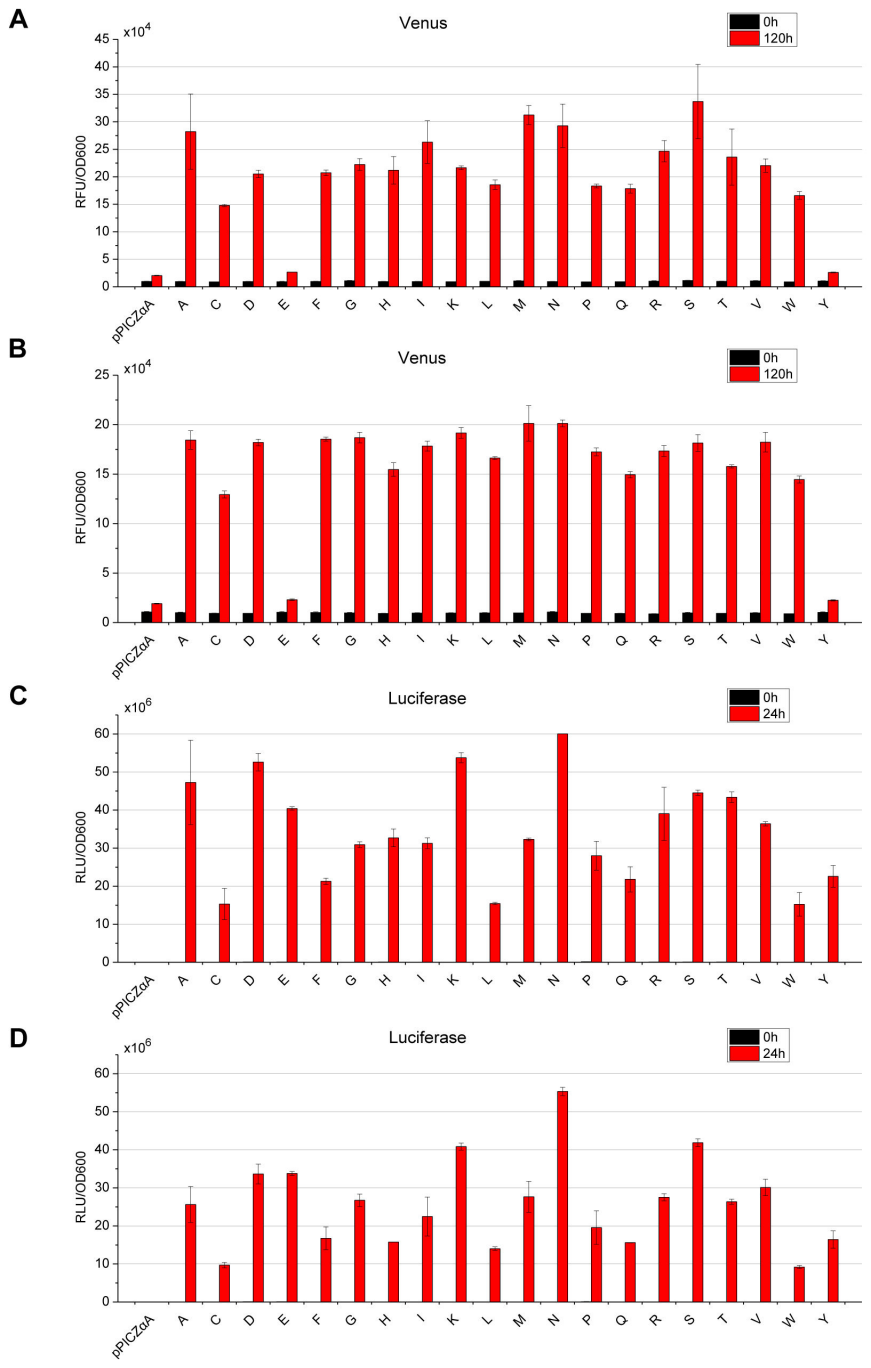
Fluorescence/luminescence measurements of Venus/luciferase libraries with variable P1’ site. Relative fluorescence/luminescence units (RFU/RLU) were normalized to the OD_600_ of the corresponding cultures and then analyzed and compared with the measurements of recombinant yeast strains with the same range of zeocin resistance. pPICZαA: empty vector control; single letters: the single-letter codes of the corresponding P1’ residues. (A) Venus library with zeocin resistance ranging 500-1,000 µg/ml and (B) 200-500 µg/ml; (C) luciferase library with zeocin resistance ranging 500-1,000 µg/ml and (D) 200-500 µg/ml. Each value representing the average of 5-6 individual colonies for each vector with the same resistance range for zeocin was presented as mean±SEM.

**Figure 3:**
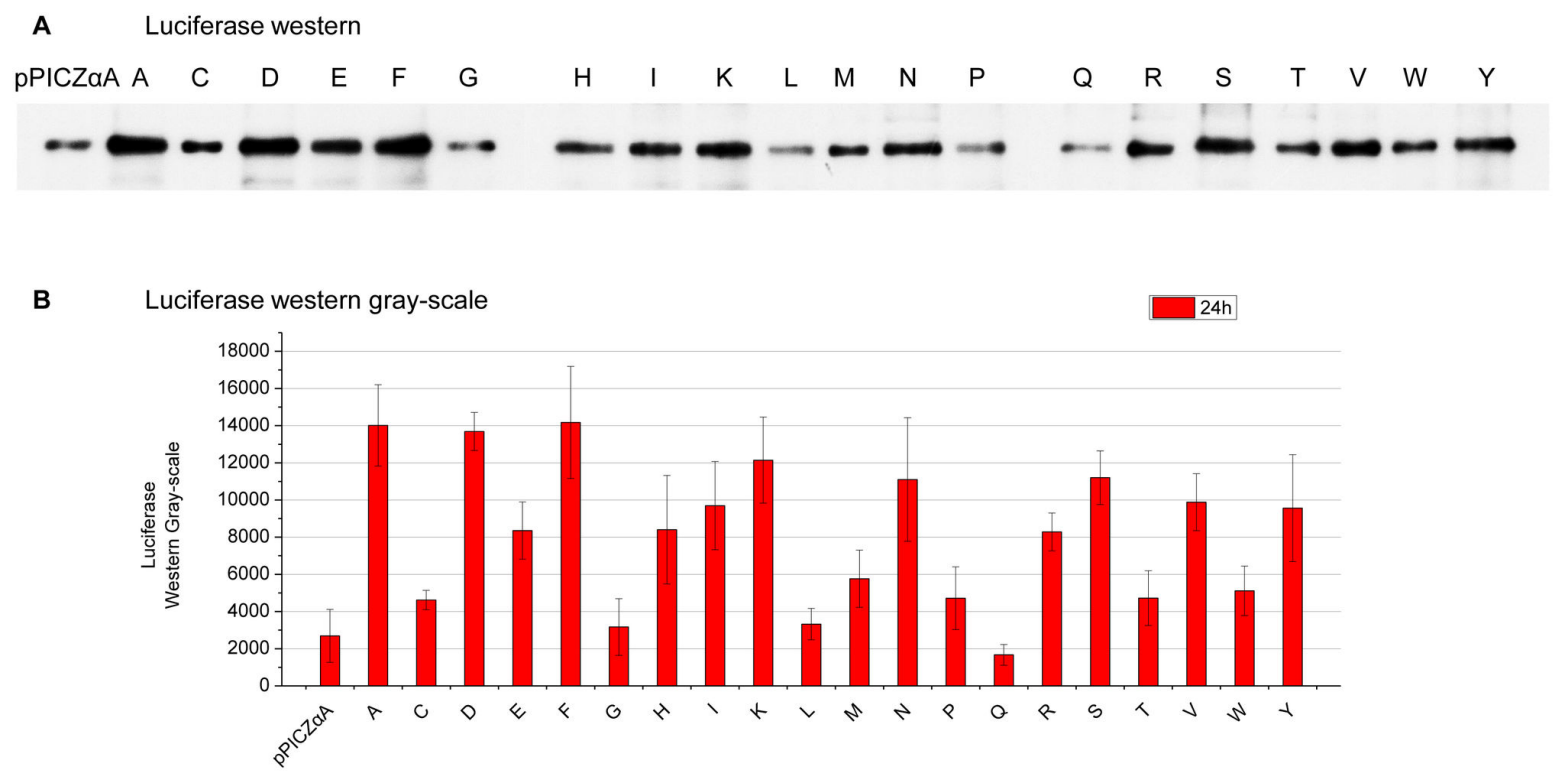
Luciferase protein determination with western blotting analysis of luciferase library. (A) A typical western result of TCA precipitated yeast culture supernatants of luciferase library with variable P1’ site, all tested recombinant yeast strains were randomly picked with zeocin resistance ranging 500-1,000 µg/ml, 5 µg of supernatant proteins were loaded for the SDS-PAGE and (B) the western gray-scale intensity of luciferase library. pPICZαA: empty vector control; single letters: the single-letter codes of the corresponding P1’ residues.

Cultivations were sampled every 24 hours till 120 hours post methanol induction. The supernatants of the yeast cultures were assayed for fluorescence (Venus) or luminescence (luciferase). As shown in Figure 2, different P1’ amino acids rendered considerable differences on the recombinant protein levels in supernatants. In particular, the highest levels in Venus library were achieved by S-Venus (serine at the P1’ site), which were nearly 13 folds higher than that of Y-Venus, the lowest ones (Figure 2A, B). In the luciferase library, the highest N-luciferase produced approximately four folds as much compared to the lowest C-luciferase (Figure 2C, D). Our western result (Figure 3) of luciferase library supported the luminescent measurements since the A, D, K, N, S-luciferase appeared to possess more secretory productivity over the others while the C, L, Q, W-luciferase did not produce much at all. This result indicated that for different proteins different P1’ site is optimal.

### Optimization of Expression for Mammalian Proteins

**Figure 4:**
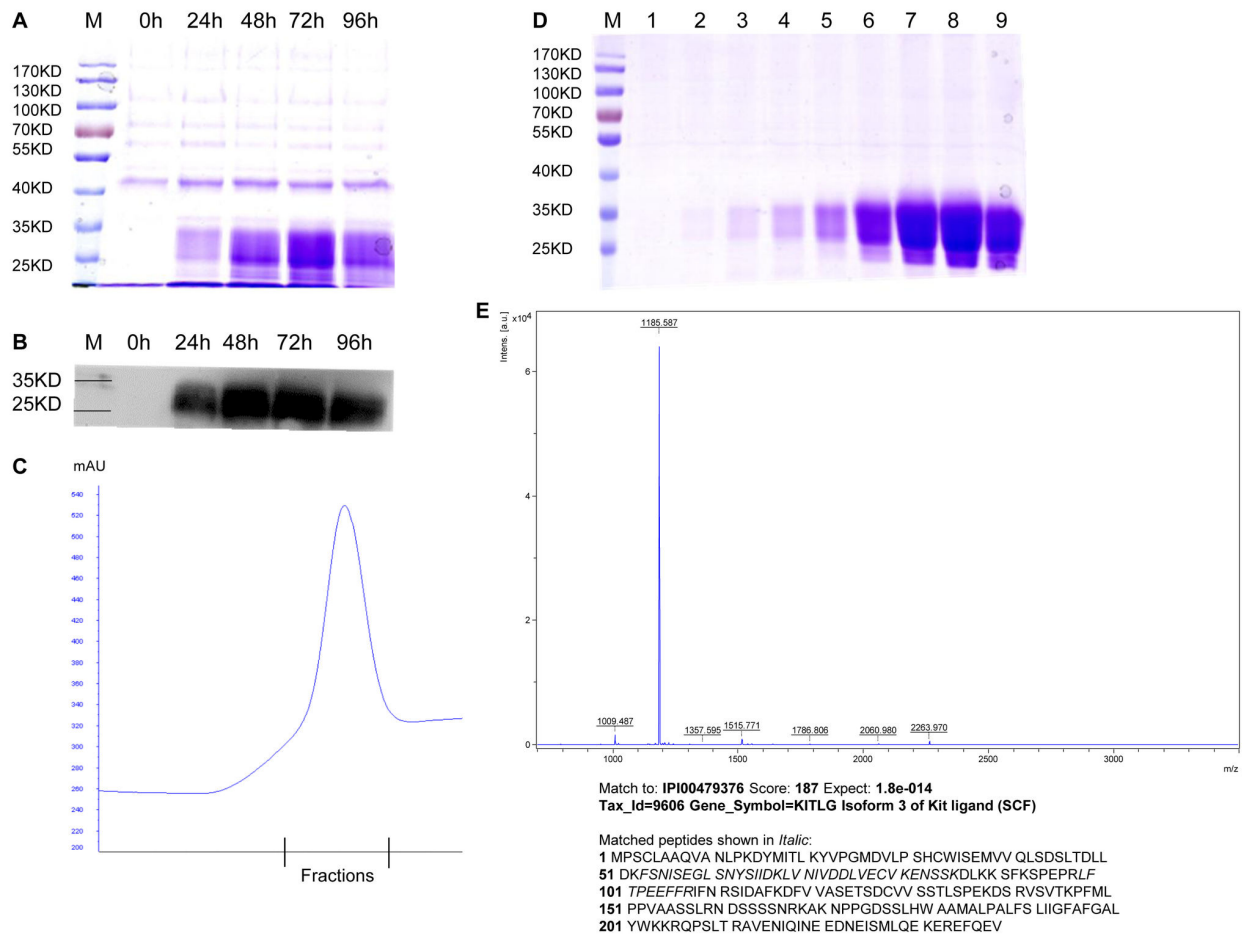
Scale-up expression, purification and mass spectrometric analysis of SCF (about 20 KDa). (A) Time course for the induction of SCF in 12% SDS-PAGE of TCA precipitated yeast culture supernatant, stained with Coomassie R-250; (B) the corresponding time course showing western blotting result; (C) Ni^2+^-HisTrap^TM^ elution profile of recombinant SCF, the bound protein was eluted with a gradient of 20-500 mM imidazole; and (D) Coomassie-stained 12% SDS-PAGE of collected fractions 1-9, 4.8 mg purified SCF in 6 ml was obtained from 400 ml yeast culture supernatant; (E) MALDI TOF/TOF^TM^ MS analysis of the purified recombinant SCF.

To achieve the objective of increasing the yields of mammalian proteins with medical relevance, several such proteins were expressed using this methodology. The results showed that the yields of all the recombinant proteins tested could be enhanced by P1’ site replacement (Table 2). The most productive stem cell factor (V-SCF) shown in Table 2 enable us to express and obtain substantial amount of recombinant SCF through a scale-up yeast fermentation and protein purification (Figure 4). The identity of the harvested recombinant protein was confirmed to be SCF by Mass Spectrometry analysis (Figure 4), and its SCF bioactivity was confirmed by tests on hematopoietic stem cells (data not shown). SCF is a well-known cytokine that plays an important role in hematopoiesis, spermatogenesis and melanogenesis [45-53], which may be used along with other cytokines to culture hematopoietic stem cells and hematopoietic progenitors [45,51]. The cultivation of such stem cells would provide sufficient hematopoietic progenitor cells in clinical bone marrow transplantation in the treatment of leukemia and other diseases. Our example (Figure 4) demonstrated that protein of great interests could be efficiently produced in large amounts with the help of our system, especially those with less secretory productivity by traditional means. As another proof of principle, we have designed a degenerative library at the P1’ site for secretory expression of previously poorly expressed human tissue plasminogen activator (tPA) and interleukin 4 (data not shown).

### Genomic Integration of Additional *Kex2* Copies

**Figure 5:**
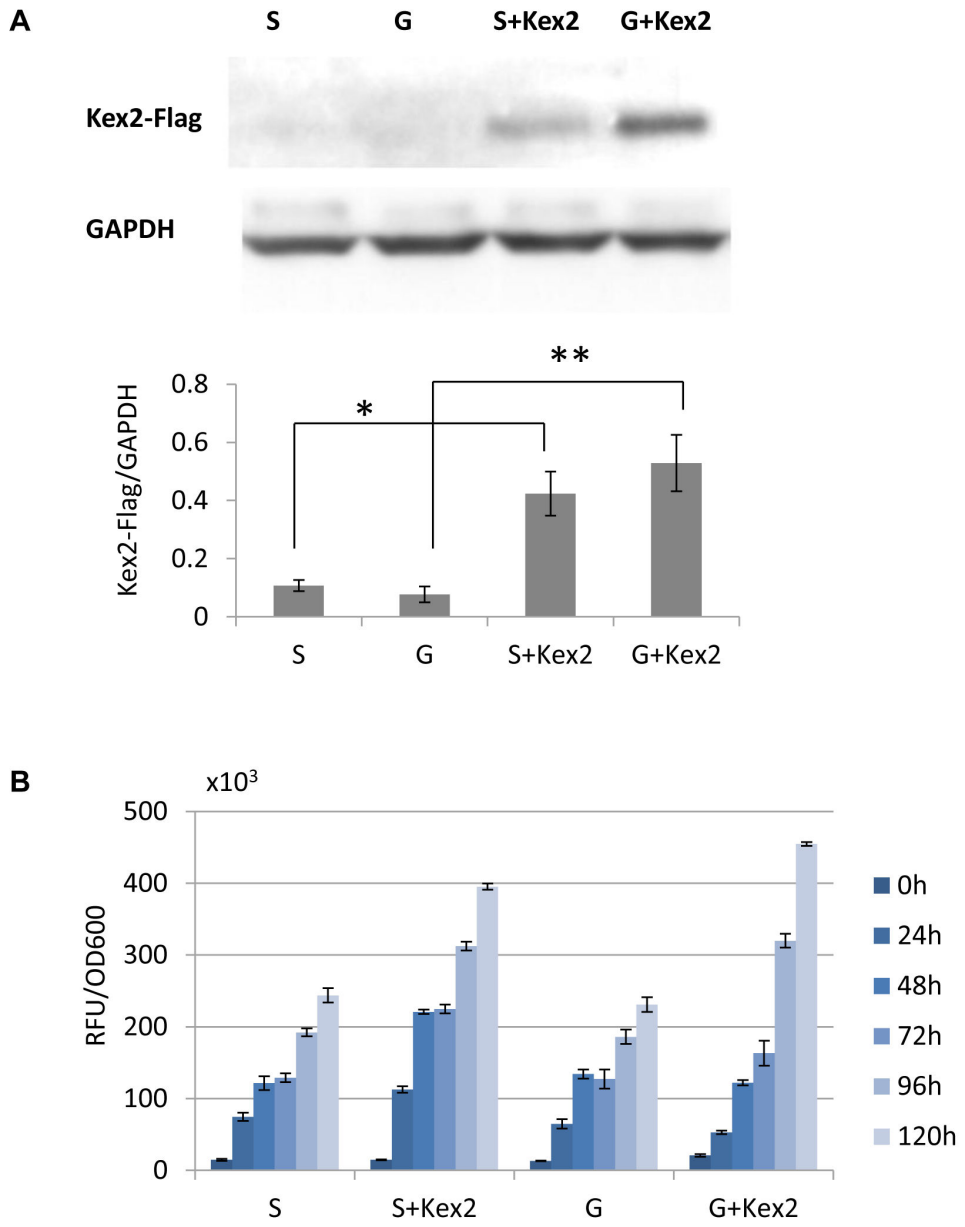
Integration of additional *Kex2* copies and the resulting influence on Venus fluorescence measurements: (A) western blotting (protein loads: 30 μg/well) showing the expression of integrated additional *Kex2* copies (with flag tag), *P* values determined by *t*-tests of and S, G (Zeo 200-500 µg/ml) are *: 0.0022 and **0.0015 respectively; and (B) comparisons of Venus fluorescence measurements (normalized to the OD_600_ of the corresponding cultivation) between recombinant strains with zeocin resistance ranging from 200-500 µg/ml. Each value representing the average of 5-6 individual colonies for each vector with the same resistance range for zeocin was presented as mean±SEM.

Finally, we investigated whether additional copies of Kex2 in *P* *. pastoris* hosts could further increase the secretory productivity. To this end, the Venus expression constructs with/without additional *Kex2* copies were chosen, cultivated, sampled and expression levels determined. Western blotting analysis showed that introduction of additional *Kex2* copies greatly increased the Kex2 expression in the yeast host cells (Figure 5A). The fluorescence intensity in yeast culture media was significantly elevated upon addition of more *Kex2* copies in that G-Venus-kex2 nearly doubling the productivity (Figure 5B). Taken together, our results clearly demonstrated that the feasibility of achieving high levels of recombinant secretory proteins in *P* *. pastoris* by optimizing P1’ site and increased Kex2 copies.

## Discussion

During decades of using *Pichia* *pastoris* as an eukaryotic protein expression system, the problem of inconsistent secretory productivity among different recombinant proteins, i.e. some proteins could reach extremely high yields [26-28] while some others had little or no expression at all, has always been a major obstacle for routine application in both research and industry. A recently published report found that the folded protein flux through the cellular secretory pathway rather than the transcription and translation was most likely the rate-limiting step to the secretory protein production event [25], based on a systematic series of analysis and mathematical simulations. However, few reports have directly addressed this issue and provided efficient ways to increase the secretory yields of recombinant proteins by improving the flux of proteins through the secretory pathway. Our present study suggested a new strategy to increase *P* *. pastoris* secretory productivity by optimizing the yeast convertase Kex2 cleavage.

Our study was carried out with the commonly used yeast secretory expression vector, i.e., pPICZαA. Through generating a library of vectors (Figure 1), yeast transformation and secretory expression assays, we found that variable P1’ site amino acid greatly influenced the recombinant proteins secretory yields (Figure 2, Figure 3, Table 2) as described in Results. Unlike previous results based on *in vitro* enzymatic data [38,54], our *in vivo* experiments might reflect the physiological situation of the *P* *. pastoris* host cells since influences of different P1’ residue on the secretory yields were variable. The most significant result of our study clearly demonstrated that the patterns and the extents of these variations depended on different proteins (Figure 2, Figure 3, Table 2). Based on this discovery, one could possibly identify the most productive P1’ amino acids for any given recombinant proteins, and maximize the secretory productivity as exemplified in Figure 4.

Other than optimization of the P1’ residues, integration of additional constitutively expressing *Kex2* copies into the *Pichia* yeast genome has also been proved to significantly improve the secretory yields of recombinant secretory proteins regardless of the P1’ residues in our study (Figure 5), which demonstrated that the Kex2 cleavage was pivotal for improving yeast secretory yields and that the improvement could be achieved by either selection of the optimum Kex2 substrates, and/or elevating the levels of Kex2 or both. In summary, the major finding of this study is to enhance the *P* *. pastoris* secretory productivity of recombinant proteins by making optimal use of Kex2 activity, which was accomplished by optimization of Kex2 P1’ site residue and/or introduction of additional *Kex2* copies into yeast genome.
